# ﻿Two new species and additional records of the genus *Asiageodromicus* Shavrin, 2024 from Xizang, southwest China (Coleoptera, Staphylinidae, Omaliinae)

**DOI:** 10.3897/zookeys.1241.150872

**Published:** 2025-06-11

**Authors:** Yi Yang, Nima Danzeng, Zhong Peng

**Affiliations:** 1 College of Life Sciences, Shanghai Normal University, 100 Guilin Road, 1st Educational Building 323 Room, Shanghai, 200234, China Shanghai Normal University Shanghai China; 2 Institute of Plateau Biology of Xizang Autonomous Region, Lhasa, Xizang Autonomous Region, 850001, China Institute of Plateau Biology of Xizang Autonomous Region Lhasa China

**Keywords:** Morphology, new record, new species, rove beetles, taxonomic key, taxonomy

## Abstract

*Asiageodromicusdawai* Yang & Peng, **sp. nov.** (Xizang: Langkazi) and *Asiageodromicuszhangi* Yang & Peng, **sp. nov.** (Xizang: Cuona) are described and illustrated. Additional records of *A.namucuoicus* (Cheng & Peng, 2020) are reported. A key to the Chinese species of the genus *Asiageodromicus* Shavrin, 2024 is given.

## ﻿Introduction

The genus *Asiageodromicus* Shavrin, 2024 (Anthophagini) includes eight species distributed in the Palaearctic and Oriental regions (Shavrin 2024, Shavrin 2025). According to a key provided by Shavrin (2024), three species of *Asiageodromicus* were reported from China: *A.amplissimus* (Shavrin, 2019) (China: Sichuan, Yunnan), *A.namucuoicus* (Cheng & Peng, 2020) (China: Xizang), and *A.subquadratus* (Cheng, Shavrin & Peng, 2020) (China: Xizang).

These three Chinese species of the genus *Asiageodromicus* were originally classified under the genus *Geodromicus* Redtenbacher, 1857, but were subsequently transferred to *Asiageodromicus* based on the more protruded latero-apical portions and deeper apical emargination of the labrum, longer and narrower antennomeres 3–10, more transverse pronotum, smaller basal portion and broadened median lobe, the lack of sclerotized structures of the internal sac, and the longer gonocoxites of the female genital segment (Shavrin 2024).

A study of *Asiageodromicus* material from Xizang yielded a new record and two new species.

## ﻿Material and methods

The genitalia and other dissected parts were mounted on plastic slides and attached to the same pin as the respective specimens. Photographs were taken with a Canon EOS 7D camera with a Canon MP-E 65 mm macro lens or with a Canon G9 camera mounted on an Olympus CX 31 compound microscope.

The following measurements are used in this paper and abbreviated as follows: **BL**—total body length (from anterior margin of clypeus to apex of abdomen); **FL**: length of forebody (from anterior margin of clypeus to apex of the elytra); **HW**—maximum width of head including eyes; **HL**—length of head (from base of labrum to posterior constriction of head); **OL**—ocular length (longitudinal); **LT**—length of temple; **AL**—length of antenna; **PL**—length of pronotum; **PWmax**—maximum width of pronotum; **PWmin**—minimum width of pronotum; **ESL**—sutural length of elytra (length of elytra from the apex of scutellum to the posterior margin of sutural angle); **EW**—maximum width of elytra; **MTbL**—length of metatibia; **MTrL**—length of metatarsus; **AW**—maximum width of abdomen; **AedL**—length of aedeagus (from base of median lobe to apex of parameres).

All material treated in this paper is deposited in the Insect Collection of Shanghai Normal University, Shanghai, China (**SNUC**). The type labels are cited using the original spelling; different labels are separated by slashes.

## ﻿Results

### ﻿ Asiageodromicus dawai

Taxon classificationAnimaliaColeopteraStaphylinidae

Yang & Peng sp. nov.

35BED532-F311-5C2D-8BD9-5E5D9C78DE1C

https://zoobank.org/57BEB2C5-64C7-4895-8397-4B5D082A645C

Figs 1A , 2 , 4–6

#### Type material.

***Holotype*.** China – **Xizang Prov.** • ♂; glued on a card with two labels as follows: “China: Xizang Prov., Langkazi Co., Puma Yumco, 28°37'17"N, 90°27'01"E, 5000 m, 02.VII.2021, Z. Peng, Z. Yin & W. Zhang leg.” “HOLOTYPE: *Asiageodromicusdawai* sp. nov., Yang & Peng des. 2025” [red handwritten label]; SNUC. ***Paratypes*.** China – **Xizang Prov.** • 6 ♂♂, 37 ♀♀; glued on the cards, each card with two labels as follows: “China: Xizang Prov., Langkazi Co., Puma Yumco, 28°37'17"N, 90°27'01"E, 5000 m, 02.VII.2021, Z. Peng, Z. Yin & W. Zhang leg;” “PARATYPE: *Asiageodromicusdawai* sp. nov., Yang & Peng des. 2025” [yellow printed label]; SNUC.

#### Description.

Measurements (in mm) and ratios: BL: 5.17–6.22; FL: 3.84–4.09; HW: 0.89–1.07; HL: 0.52–0.65; OL: 0.22–0.33; LT: 0.09–0.15; AL: 2.61–3.13; PL: 0.74–0.91; PWmax: 1.20–1.33; PWmin: 1.04–1.22; ESL: 1.39–1.70; EW: 1.87–2.12; MTbL: 0.99–1.11; MTrL: 0.30–0.43; AW: 1.80–2.10; AedL: 1.23–1.27.

**Figure 1.**
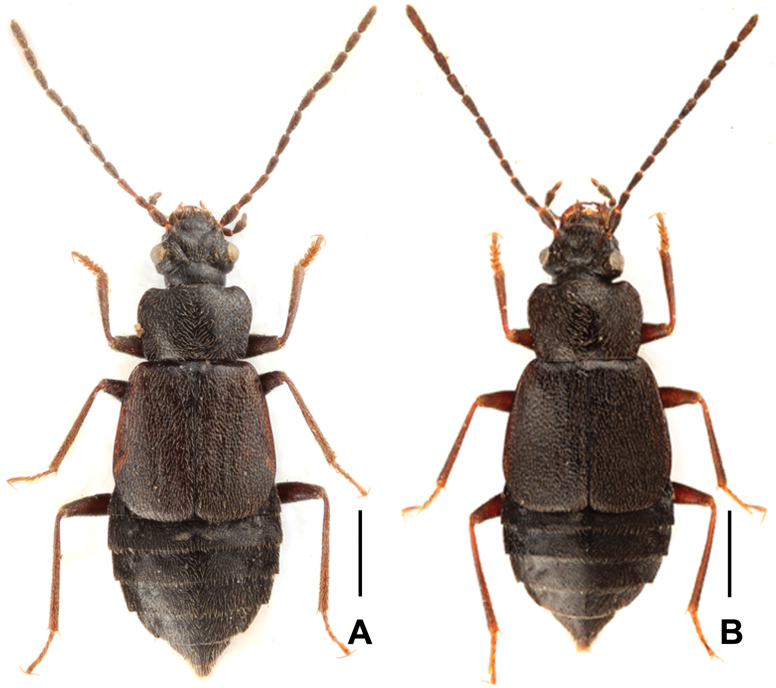
Habitus **A***Asiageodromicusdawai* sp. nov. **B***Asiageodromicuszhangi* sp. nov. Scale bars: 1.0 mm.

Habitus as in Fig. 1A. Body blackish-brown, with paler, reddish-brown elytra and apical abdominal tergites; legs brown; mouthparts, antennae, and tarsi light brown. Head with fine microsculpture; neck with distinct large isodiametric sculpture; pronotum with distinct isodiametric microreticulation; elytra and scutellum without microsculpture; abdominal tergites with dense and distinct isodiametric microsculpture.

Head transverse, convex between anteromedian depression and in portions between ocelli and eyes; frontal portion with relatively deep and wide anteromedian depression, distinctly narrowed basad to level of anterior margins of eyes; interocellar depression moderately deep, slightly narrowing basad, separated from infraorbital ridges by fine and long convergent grooves in front of ocelli; temples convex, less than half as long as eyes. Eyes moderately large, strongly convex. Ocelli small, distance between ocelli about 1.3–2.0 times as long as distance between ocellus and posterior margin of eye. Punctation irregular and fine, indistinct in apical portion, more distinct and denser on infraorbital ridges. Neck with indistinct and irregular fine punctures. Preapical segment of maxillary palp about 0.8 times as long as preceding segment, markedly widened apicad; apical palpomere about 0.7 times as long as preapical segment, widest in basal portion, gradually narrowing apically. Antennae moderately long, exceeding apical third of elytra when reclined; lengths × widths of antennomeres (holotype): 1: 0.26 × 0.11; 2: 0.17 × 0.09; 3: 0.26 × 0.09; 4: 0.24 × 0.07; 5: 0.24 × 0.09; 6–7: 0.54 × 0.11; 8–9: 0.52 × 0.14; 10: 0.24 × 0.13; 11: 0.35 × 0.09.

Pronotum slightly convex, transverse, widest in anterior third, narrower posteriad than anteriad, with anterior angles rounded and indistinctly protruding; narrow basal part of pronotum with straight lateral margins and with obtuse posterior angles; median portion without impressions. Punctation dense, markedly larger, deeper, and coarser than that in head, finer and sparser in middle.

Elytra slightly convex, slightly wider than long, widened posteriad. Punctation as that in pronotum or slightly shallower, markedly finer and denser on prescutellar portion. Scutellum with fine, irregular punctures.

Abdomen slightly broader or about as wide as elytra, convex, with two large, transverse tomentose spots in the middle of tergite IV. Punctation distinct, very dense and fine.

**Figure 2.**
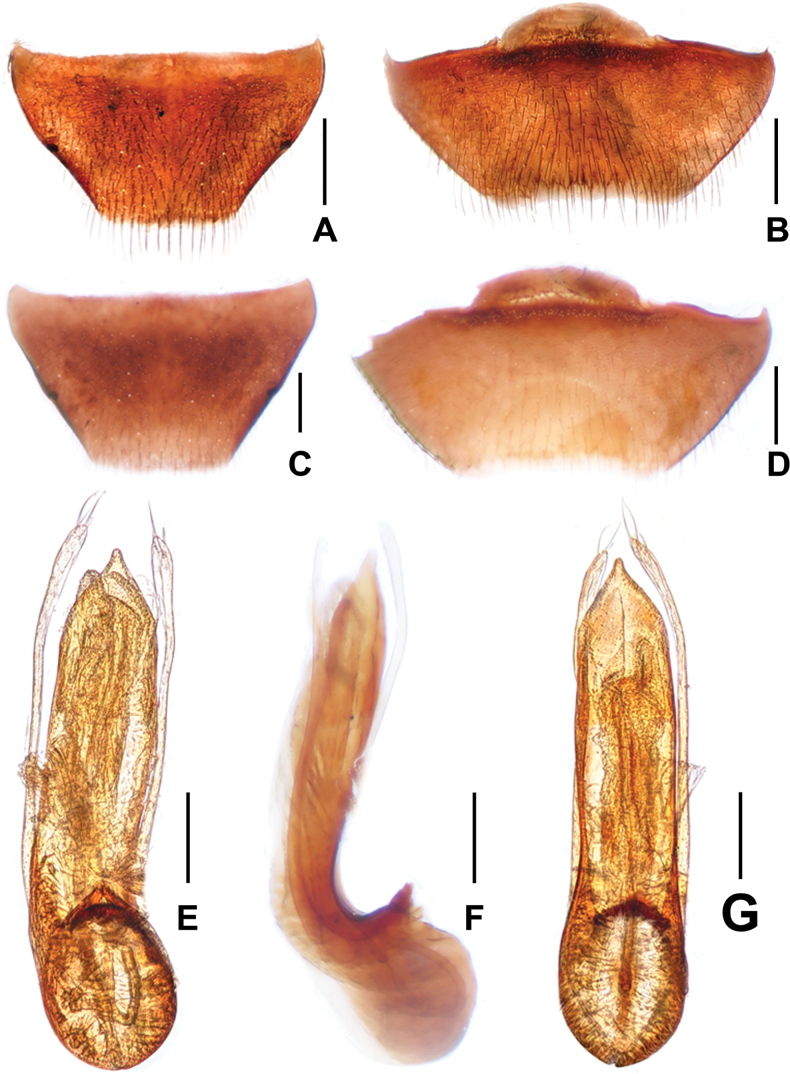
*Asiageodromicusdawai* sp. nov. **A** female tergite VIII **B** female sternite VIII **C** tergite VIII **D** male sternite VIII **E** aedeagus in ventral view **F** aedeagus in lateral view **G** aedeagus in dorsal view. Scale bars: 0.2 mm.

**Male.** Protarsomeres 1–4 markedly wide. Apical margin of abdominal tergite VIII (Fig. 2C) nearly truncate; apical margin of abdominal sternite VIII (Fig. 2D) broadly concave. Aedeagus (Fig. 2E–G) with small basal portion rotated inside abdomen in lateral position (90°, when viewed dorsally); long median lobe, gradually narrowed toward apex; parameres narrow, slightly curved in apical portion, extending beyond apex of median lobe, each bearing two long and two short apical setae; internal sac weakly sclerotized, with very long flagellum coiled in basal part of aedeagus.

**Female.** Protarsomeres 1–4 narrow. Apical margins of abdominal tergite VIII and sternite VIII truncate.

#### Distribution and biological notes.

The type locality is situated to the south of Langkazi, southern Xizang. The specimens were collected at an elevation of 5000 m by sifting mixed leaf litter and sand or from under stones near Pumuyongcuo Lake (Figs 4–6).

#### Etymology.

This species is dedicated to Mr Dawa, who supported us on our field trips.

#### Comparative notes.

Based on the similar external and sexual characters, particularly the similar structure of the aedeagus, *A.dawai* is most similar to *A.amplissimus* Shavrin, 2019. Externally, *A.dawai* differs from *A.amplissimus* by the somewhat smaller body size, shorter antenna and elytra, with less dense punctation of pronotum and smaller tomentose spots in the middle of tergite IV. For illustrations of *A.amplissimus* see Shavrin (2019: figs 2, 8–9).

### ﻿ Asiageodromicus zhangi

Taxon classificationAnimaliaColeopteraStaphylinidae

Yang & Peng sp. nov.

6A56F59C-493C-5DF4-BB10-4991737F2B25

https://zoobank.org/E880196B-E2D2-4CEE-A6BD-DBA7D5693736

Figs 1B , 3 , 7

#### Type material.

***Holotype*.** China – **Xizang Prov.** • ♂; glued on a card with two labels as follows: “China: Xizang Prov., Cuona County, Lebu Valley, 27°55'13"N, 91°51'16"E, 3650–4050 m, 05.VII.2021, Peng, Yin & Zhang leg.” “HOLOTYPE: *Asiageodromicuszhangi* sp. nov., Yang & Peng des. 2025” [red handwritten label]; SNUC. ***Paratypes*.** China – **Xizang Prov.** • 3 ♂♂, 3 ♀♀; glued on the cards, each card with two labels as follows: “China: Xizang Prov., Cuona County, Lebu Valley, alt. 27°55'13"N, 91°51'16"E, 3650–4050 m, 05.VII.2021, Peng, Yin & Zhang leg;” “PARATYPE: *Asiageodromicuszhangi* sp. nov., Yang & Peng des. 2025” [yellow printed label]; SNUC.

#### Description.

Measurements (in mm) and ratios: BL: 5.55–7.10; FL: 4.03–4.54; HW: 1.13–1.20; HL: 0.63–0.73; OL: 0.23–0.30; LT: 0.13–0.18; AL: 3.30–3.55; PL: 0.95–1.05; PWmax: 1.43–1.58; PWmin: 1.23–1.40; ESL: 1.45–1.70; EW: 2.13–2.38; MTbL: 1.23–1.28; MTrL: 0.28–0.38; AW: 2.15–2.50; AedL: 1.35–1.37.

Habitus as in Fig. 1B. Body blackish-brown, with slightly paler elytra; legs reddish-brown; mouthparts and antennae light brown. Head with fine microsculpture; neck with distinct isodiametric sculpture; pronotum with distinct isodiametric microreticulation; elytra and scutellum without microsculpture; abdominal tergites with dense and fine isodiametric microreticulation.

Head transverse, convex between anteromedian depression and in portions between ocelli and eyes; frontal portion with slightly elevated supra-antennal protuberance, with two relatively deep and round median depression, distinctly narrowed basad to level of anterior margins of eyes; interocellar depression relatively deep, unseparated, slightly narrowing basad; temples convex, less than half as long as eyes. Eyes moderately large, strongly convex. Ocelli small, distance between ocelli about 1.1–1.6 times as long as distance between ocellus and posterior margin of eye. Punctation irregular and fine, distinct in apical portion. Neck with indistinct and regular fine punctures. Preapical segment of maxillary palp about 0.9 times as long as preceding segment, markedly widened apicad; apical palpomere about 0.8 times as long as preapical segment, widest in basal portion, gradually narrowing apically. Antennae moderately long, exceeding apical third of elytra when reclined; lengths × widths of antennomeres (holotype): 1: 0.35 × 0.15; 2: 0.28 × 0.10; 3: 0.28 × 0.10; 4: 0.30 × 0.10; 5: 0.28 × 0.10; 6–7: 0.60 × 0.13; 8–9: 0.63 × 0.10; 10: 0.28 × 0.16; 11: 0.45 × 0.16.

Pronotum slightly convex, transverse, widest in anterior twice, narrower posteriad than anteriad, with anterior angles rounded and indistinctly protruding; narrow basal part of pronotum with straight lateral margins and with obtuse posterior angles; median portion without impressions. Punctation dense, markedly smaller, shallow, and coarser than that in head, finer and sparser in middle.

Elytra slightly convex, slightly wider than long, slightly widened posteriad. Punctation markedly deeper, coarser and denser on prescutellar portion. Scutellum with fine, irregular punctures.

Abdomen broader than elytra, convex, with two medium-sized, transverse tomentose spots in the middle of tergite IV. Punctation distinct, very dense and fine.

**Figure 3.**
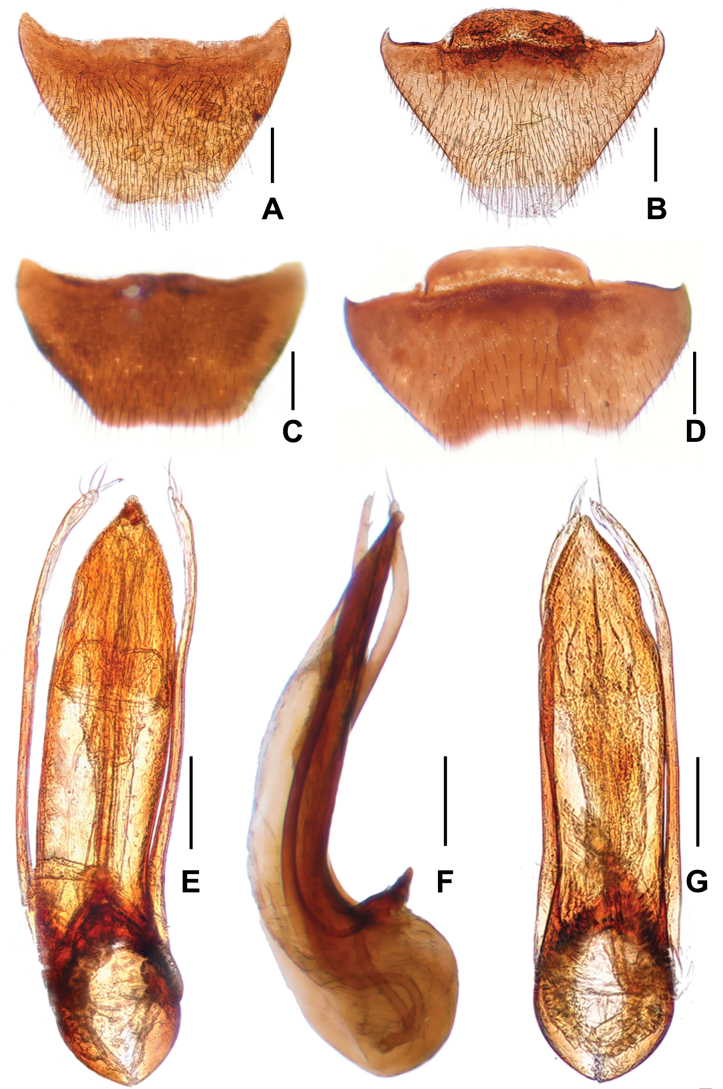
*Asiageodromicuszhangi* sp. nov. **A** female tergite VIII **B** female sternite VIII **C** tergite VIII **D** male sternite VIII **E** aedeagus in ventral view **F** aedeagus in lateral view **G** aedeagus in dorsal view. Scale bars: 0.2 mm.

**Male.** Protarsomeres 1–4 markedly wide. Apical margins of abdominal tergite VIII (Fig. 3C) weakly concave; apical margins of abdominal sternite VIII (Fig. 3D) broadly concave. Aedeagus (Fig. 3E–G) with moderately large basal portion; relatively wide and long median lobe, gradually narrowed toward apex; parameres narrow, curved in apical portion, almost reaching apex of median lobe, each bearing three long and one short apical setae; internal sac sclerotized, with very long flagellum coiled in basal part of aedeagus.

**Female.** Protarsomeres 1–4 narrow. Apical margins of abdominal tergite VIII truncate; Apical margins of abdominal sternite VIII slightly convex.

#### Distribution and biological notes.

The type locality is situated to the southwest of Cuona, southern Xizang. The specimens were collected at elevations from 3650 to 4050 m by sifting moss and litter near banks of streams (Fig. 7).

**Figures 4–7.**
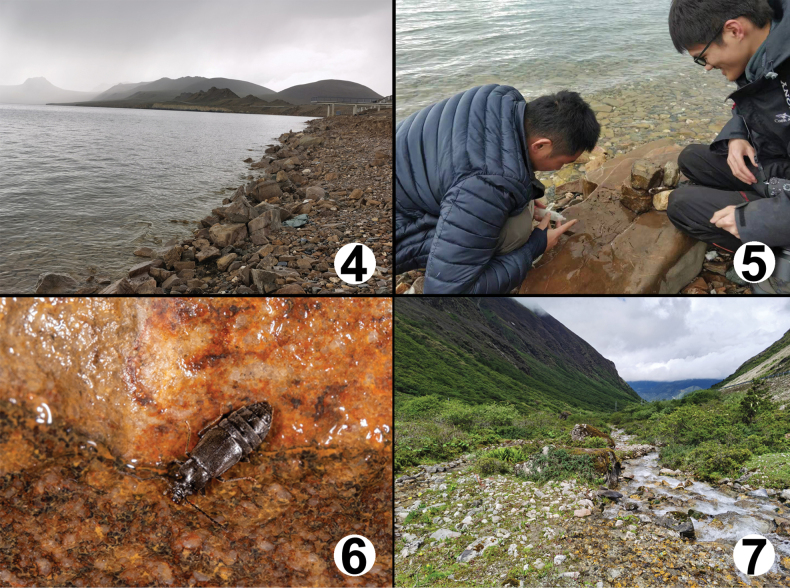
Collection and habitat of *Asiageodromicuszhangi* sp. nov. **4** habitat of *A.dawai* (Pumuyongcuo Lake) **5** Wen-Xuan Zhang (right) and Zhong Peng (left) collecting *A.dawai***6***A.dawai* was found walking under the stone **7** habitat of *A.zhangi* (Lebu Valley).

#### Etymology.

The species is dedicated to Mr Wen-Xuan Zhang, who is one of the collectors of the type specimens.

#### Comparative notes.

Based on the similarly derived morphology of the aedeagus (relatively wide and long median lobe, narrow and very long flagellum in internal sac), as well as the similar shape of the male tergite VIII, *A.zhangi* is most similar to *A.subquadratus* (Cheng, Shavrin & Peng, 2020). Externally, *A.zhangi* differs from *A.subquadratus* by shorter elytra, unseparated interocellar depression, smaller tomentose spots in the middle of tergite IV, with no spots on tergite V, and by the posteriorly more broadly concave male sternite VIII. For illustrations of *A.zhangi* see Figs 1B, 3 and for *A.subquadratus* see Cheng et al. (2020: figs 95, 97–101).

### ﻿ Asiageodromicus namucuoicus

Taxon classificationAnimaliaColeopteraStaphylinidae

(Cheng & Peng, 2020)

8DA1B410-3919-5914-B47B-0C2FE8FB7020

#### Material examined.

China – **Xizang** • 1 ♀; Dangxiong Co., near Namucuo Lake, alt. 4700 m, 02.VIII.2022, Peng, Yin & Zhang leg; SNUC.

#### Comment.

The original description of *Asiageodromicusnamucuoicus* Cheng & Peng, 2020 is based on six type specimens from “China: Xizang A. R., Lasa City, Dangxiong County, near Namucuo Lake” (Cheng et al. 2020). For illustrations of the male sexual characters see Cheng et al. (2020: figs 57, 59–63).

##### ﻿Key to the Chinese species of genus *Asiageodromicus* Shavrin, 2024

**Table:** 

1	Elytra short, markedly less than twice as long as pronotum	**2**
–	Elytra longer, twice or more than twice as long as pronotum	**4**
2	Body reddish-brown to brown. Head 1.2 times as wide as long. Apical margin of male sternite VIII slightly sinuate. China (Xizang)	***A.namucuoicus* (Cheng & Peng, 2020)**
–	Body blackish-brown. Head 1.5–1.8 times as wide as long. Apical margin of male sternite VIII broadly concave	**3**
3	Pronotum narrower (PWmax: 1.20–1.33). Aedeagus shorter (AedL: 1.23–1.27), with narrow median lobe. China (Xizang)	***A.dawai* sp. nov.**
–	Pronotum wider (PWmax: 1.43–1.58). Aedeagus longer (AedL: 1.35–1.37), with moderately wide median lobe. China (Xizang)	***A.zhangi* sp. nov.**
4	Head 1.2 times as wide as long. Antennae shorter (AL: 3.40). China (Xizang)	***A.subquadratus* (Cheng, Shavrin & Peng, 2020)**
–	Head 1.6–1.9 times as wide as long. Antennae longer (Al ≥ 3.50). China (Sichuan, Yunnan)	***A.amplissimus* (Shavrin, 2019)**

## Supplementary Material

XML Treatment for Asiageodromicus dawai

XML Treatment for Asiageodromicus zhangi

XML Treatment for Asiageodromicus namucuoicus

## ﻿Additional information

### Author ORCIDs

Yi Yang https://orcid.org/0000-0003-1132-9103

Zhong Peng https://orcid.org/0000-0001-5959-1536

### Data availability

All of the data that support the findings of this study are available in the main text.
